# Sodium Thiosulfate Reduces Acute Kidney Injury in Patients Undergoing Cytoreductive Surgery Plus Hyperthermic Intraperitoneal Chemotherapy with Cisplatin: A Single-Center Observational Study

**DOI:** 10.1245/s10434-021-10508-x

**Published:** 2021-08-04

**Authors:** Annika Kurreck, Felix Gronau, Miguel Enrique Alberto Vilchez, Wiltrud Abels, Philipp Enghard, Andreas Brandl, Roland Francis, Bettina Föhre, Christian Lojewski, Johann Pratschke, Peter Thuss-Patience, Dominik Modest, Beate Rau, Linda Feldbrügge

**Affiliations:** 1 Department of Hematology, Oncology and Tumorimmunology, Charité – Universitätsmedizin Berlin, Corporate Member of Freie Universität Berlin and Humboldt-Universität zu Berlin, Berlin, Germany; 2 Department of Surgery, Charité – Universitätsmedizin Berlin, Corporate Member of Freie Universität Berlin and Humboldt-Universität zu Berlin, Berlin, Germany; 3 Department of Anesthesiology and Operative Intensive Care Medicine, Charité – Universitätsmedizin Berlin, Corporate Member of Freie Universität Berlin and Humboldt-Universität zu Berlin, Berlin, Germany; 4 Department of Nephrology and Medical Intensive Care, Charité – Universitätsmedizin Berlin, Corporate Member of Freie Universität Berlin and Humboldt-Universität zu Berlin, Berlin, Germany; 5 Digestive Unit, Champalimaud Foundation, Lisbon, Portugal

## Abstract

**Background:**

Cytoreductive surgery (CRS) in combination with hyperthermic intraperitoneal chemotherapy (HIPEC) represents a multimodal treatment concept for patients with peritoneal surface malignancies. The use of intraperitoneal cisplatin (CDDP) is associated with a risk of acute kidney injury (AKI). The aim of this study is to evaluate the protective effect of perioperative sodium thiosulfate (STS) administration on kidney function in patients undergoing CRS and CDDP-based HIPEC.

**Patients and Methods:**

We retrospectively analyzed clinical data of all patients who underwent CRS and CDDP-based HIPEC at our hospital between March 2017 and August 2020. Patients were stratified according to the use of sodium thiosulfate (STS vs. no STS). We compared kidney function and clinical outcome parameters between both groups and determined risk factors for postoperative AKI on univariate and multivariate analysis. AKI was classified according to acute kidney injury network (AKIN) criteria.

**Results:**

Of 238 patients who underwent CRS and CDDP-based HIPEC, 46 patients received STS and 192 patients did not. There were no significant differences in baseline characteristics. In patients who received STS, a lower incidence (6.5% vs. 30.7%; *p* = 0.001) and severity of AKI (*p* = 0.009) were observed. On multivariate analysis, the use of STS (OR 0.089, *p* = 0.001) remained an independent kidney-protective factor, while arterial hypertension (OR 5.283, *p* < 0.001) and elevated preoperative urea serum level (OR 5.278, *p* = 0.032) were predictors for postoperative AKI.

**Conclusions:**

The present data suggest that STS protects patients from AKI caused by CRS and CDDP-based HIPEC. Further prospective studies are needed to validate the benefit of STS among kidney-protective strategies.

**Supplementary Information:**

The online version contains supplementary material available at 10.1245/s10434-021-10508-x.

The efficacy and safety of cytoreductive surgery (CRS) and hyperthermic intraperitoneal chemotherapy (HIPEC) as part of a multimodal therapeutic approach in the adjuvant and palliative setting of peritoneal malignancies is the subject of current research.^1^

Cisplatin (CDDP) represents one of the most frequently used intraperitoneal chemotherapeutic agents. CDDP efficacy is enhanced by heat.^2^ Furthermore, higher doses of CDDP can be reached in the peritoneal layer with intraperitoneal compared to systemic administration.^3^ Common adverse effects of CDDP include nausea and vomiting, myelosuppression, polyneuropathy, ototoxicity, and nephrotoxicity. In this context, the incidence of acute kidney injury (AKI) following CDDP-based HIPEC is reported to be up to 20%,^4^–^8^ resulting in a prolonged hospital stay and a higher rate of severe morbidities in affected patients.^5^

Sodium thiosulfate (STS) is a water-soluble thiol compound with reducing properties, forming a nontoxic complex with CDDP that is more efficiently eliminated than protein-bound CDDP and was shown to reduce CDDP-induced ototoxicity in children.^9^^,^^10^ Intravenously administered STS was found to be highly concentrated in the kidneys. For the purpose of kidney protection, it was first concurrently administered in adult ovarian cancer patients receiving a high-dose systemic chemotherapy with CDDP.^11^

Based on the assumption that STS may also decrease the incidence of AKI in patients receiving intraperitoneal CDDP, it has been used in several clinical HIPEC trials; however, the effects of STS on the incidence and severity of AKI were not examined systematically.^12^–^14^ Recently, Laplace et al. prospectively evaluated the potential of STS in the prevention of AKI in a small patient cohort undergoing CRS and CDDP-based HIPEC. The authors found STS to significantly reduce the rate of AKI.^15^ To our knowledge, this is the only study evaluating the kidney-protective potential of STS in patients undergoing CRS and CDDP-based HIPEC by directly comparing with a group of patients not receiving STS. A remaining question is whether severity of postoperative AKI can be influenced, and whether the protective effect of STS can be reproduced in a larger patient cohort.

Based on the aforementioned findings indicating that STS represents a potent kidney-protective drug, we administered STS in all patients receiving CRS and CDDP-based HIPEC in our department starting in November 2019. The aim of the underlying retrospective analysis is to evaluate the kidney-protective potential of STS in a large patient cohort undergoing CRS and CDDP-based HIPEC by comparison with a control group. As this investigation is unplanned and not randomized, the results should be interpreted as hypothesis generating.

## Patients and Methods

### Patients

We retrospectively analyzed the clinical data of patients who underwent cytoreductive surgery (CRS) in combination with hyperthermic intraperitoneal chemotherapy (HIPEC) including cisplatin (CDDP) in the Department of Surgery, Campus Charité Mitte and Campus Virchow-Klinikum, Charité Universitätsmedizin Berlin, between November 2017 and August 2020.

All patients provided written informed consent to the collection of personal and medical data as well as its use for research purposes, according to the approval by the Charité Institutional Review Board (EA1/009/16). The data collected were stored and processed according to the *General Data Protection Regulation* and local data protection laws. The retrospective study was conducted in accordance with the ethical standards of the Helsinki Declaration of 1975.

To evaluate the kidney-protective potential of sodium thiosulfate (STS), we compared patients receiving STS with a historical control group of patients not receiving STS and consecutively analyzed both groups with regard to tumor [entity, preoperative therapies, peritoneal cancer index] and patient characteristics [age, sex, comorbidities, physical status according to the American Society of Anesthesiologists (ASA), preoperative renal function] as well as perioperative characteristics [completeness of cytoreduction (CCR), duration of surgery and duration of HIPEC, intraperitoneal chemotherapy and its dose] and postoperative outcome parameters [postoperative renal function, rate of hemodialysis, intensive care unit (ICU) length of stay (LOS) and hospital LOS, postoperative complications].

### Definition of Perioperative Characteristics and Postoperative Outcome Parameters

We evaluated pre- and postoperative renal function by reviewing the following laboratory values prior to the intervention and during the hospital stay until the patient’s discharge: serum creatinine [mg/dl], estimated glomerular filtration rate [ml/min], and serum urea [mg/dl]. Furthermore, we reviewed medical data for renal replacement therapy following CRS and HIPEC. AKI was defined as a minimum increase of serum creatinine by 0.3 mg/dl or 150–200% (1.5- to 2-fold) from baseline value in a 48-h period corresponding to stage 1 based on the classification of the Acute Kidney Injury Network (AKIN).^16^ AKIN stage 2 was defined as an increase of serum creatinine by more than 200–300% (> 2- to 3-fold). AKIN stage 3 corresponded to an increase of serum creatinine by more than 300% (> 3-fold) or a maximum creatinine of at least 4.0 mg/dl or the need of renal replacement therapy (RRT).^16^ Urine output data were not available; therefore, the definition of AKI was entirely based on serum creatinine.

Peritoneal tumor burden was assessed using the Peritoneal Cancer Index (PCI).^17^ Completeness of cytoreduction was defined according to Sugarbaker et al. as follows: no residual peritoneal lesions (CCR = 0), persisting nodules < 2.5 mm in size (CCR = 1), nodules between 2.5 mm and 2.5 cm (CCR = 2), and nodules > 2.5 cm or confluent unresectable tumor nodules (CCR = 3).^18^ The severity of postoperative complications was categorized according to the Clavien–Dindo classification.^19^ ICU LOS was measured from the day of surgery until the patient’s release from ICU, and hospital LOS was measured from the day of the intervention until the patient’s release from hospital.

For the univariable and the multivariable analysis of potential risk factors for postoperative AKI, we selected cut-offs for the continuous variables of interest as follows: PCI > 15, age > 60 years, body mass index (BMI) > 25 kg/m^2^ (definition of overweight and obesity), duration of surgery > 400 min, serum creatinine > 1.0 m g/dl, serum urea > 45 mg/dl, and estimated glomerular filtration rate (eGFR) < 90 ml/min. The cut-offs for the laboratory values were chosen according to the reference values of the laboratory competent for the analysis of our blood samples.

### Cytoreductive Surgery (CRS) and Hyperthermic Intraperitoneal Chemotherapy (HIPEC)

In all of the analyzed patients with peritoneal surface malignancy, the indication for CRS and HIPEC was confirmed by our multidisciplinary tumor board. Patients were preoperatively examined according to the in-house standards for comorbidities that may increase perioperative risk.

Surgery was performed by a small team of specialized surgeons according to our standard operating procedures (SOP) that are developed and regularly revised in a multidisciplinary consortium. In short, following a diagnostic laparoscopy to rule out potential contraindications, a long midline incision and parietal peritonectomy were performed. Further cytoreduction included different organ resections with a substantial variation of the extent of resection.^20^ Subsequent to CRS, inflow and outflow tubes were inserted into the abdomen, and HIPEC was performed after closure of the abdominal wall.

Cisplatin was administered at a dose of 75 mg/m^2^ of body surface area with either doxorubicin (15 mg/m^2^ of body surface area) or mitomycin C (15 mg/m^2^ of body surface area), using an automatic hyperthermic chemotherapy perfusion device. Chemotherapeutic agents were dissolved in heated saline solution resulting in a total volume of 3–4 l chemotherapy-containing solution. HIPEC was performed at a flow rate of 400–600 ml/min for 60 min until December 2018. Starting from January 2019, the total duration of HIPEC was increased to 90 min according to the protocols used by van Driel et al. and Verwaal et al.^12^^,^^21^ The temperature of the chemotherapeutic solution in the abdomen was kept at 43.0 ± 0.5 °C and continuously monitored. At the end of perfusion, the remaining fluid was drained without subsequent rinsing.

The postoperative care was strictly governed by our above-mentioned SOP that are guided by current evidence-based recommendations on faster recovery after surgery. This includes restrictive fluid management, optimized pain medication, early mobilization, and early food intake, among other measures.

### Administration of Sodium Thiosulfate (STS)

We administered STS in accordance with the protocol published by van Driel et al. starting in November 2019.^12^ Patients received an STS bolus injection with a dose of 9 g/m^2^ of body surface area prior to HIPEC and a continuous application of STS at a dose of 12 g/m^2^ of body surface area over 6 h following HIPEC. Serum sodium concentrations were closely monitored every 1–2 h for the entire duration of continuous STS administration. The need for dose reduction or discontinuation was consistently reevaluated based on the respective serum sodium concentrations. In the case of serum sodium exceeding 155 mmol/l following STS bolus injection during HIPEC, continuous administration of STS was not performed. In patients with serum sodium concentrations between 150 and 155 mmol/l, dose of continuous STS administration was reduced by 50% to a total of 6 g/m^2^. In all other cases, full dose of 12 g/m^2^ was administered.

### Statistical Analysis

All statistical analyses were performed using SPSS version 25.0 software (IBM Corporation, Armonk, NY, USA). In univariable analysis, categorial variables are presented as numbers (percentages) and were compared using *χ*^2^ test; continuous variables are presented as medians (ranges) and were compared using nonparametric Mann–Whitney U test or analysis of variance (ANOVA). Binary logistic regression was used for multivariate analysis. All variables demonstrating a difference between the analyzed groups *p* < 0.1 were included. The two-sided significance level was set to 0.05 with a 95% confidence interval.

## Results

### Patient and Tumor Characteristics

**Fig. 1.**
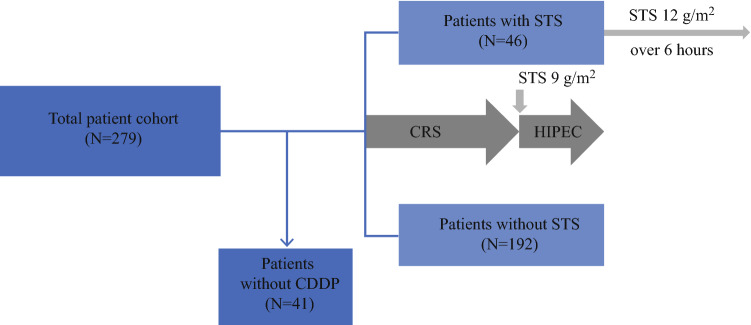
Consort diagram of analyzed patient cohort. *CDDP* cisplatin, *STS* sodium thiosulfate, *CR* cytoreductive surgery, *HIPEC* hyperthermic intraperitoneal chemotherapy

We identified 279 adult patients with peritoneal surface malignancies who underwent CRS and HIPEC in our department between November 2017 and August 2020. We excluded 41 patients from analysis who did not receive intraperitoneal CDDP. In the final analysis, 46 patients received STS between November 2019 and August 2020. In 192 patients, CRS and HIPEC were performed without the administration of STS (no STS) between November 2017 and November 2019. Please refer to Fig. 1 for a diagram illustrating the analyzed patient cohort.

**Fig. 2.**
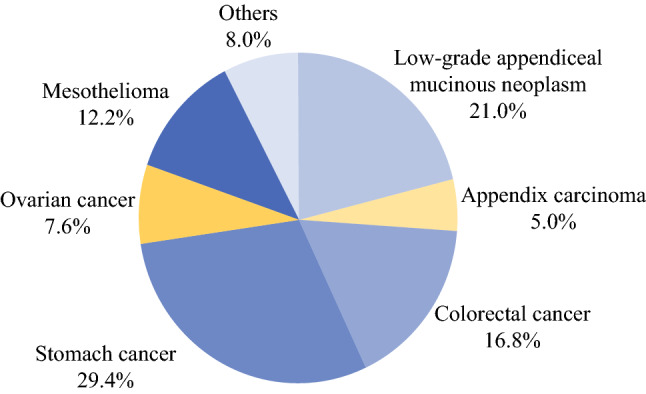
Distribution of tumor entities within the analyzed patient cohort

The median age of the analyzed patient cohort was 57 years, with a higher percentage of female patients (55.9% female). The most common tumor entities were gastric cancer followed by low-grade appendiceal mucinous neoplasm (LAMN) and colorectal cancer. Please see Fig. 2 illustrating the distribution of tumor entities within the analyzed patient cohort.

There were no significant differences in baseline characteristics between patients receiving STS and those not receiving STS (no STS). Of note, there were 14 patients (7.3%) with preexistent renal disease in the no STS group as compared with no patient in the STS group (not significant). Please refer to Table 1 for detailed information on baseline characteristics of all patients and patients separated according to the administration of STS.

**Table 1:** Baseline and intraoperative characteristics

	All patients (*N* = 238)	No STS (*N* = 192)	STS (*N* = 46)	*p* value
Age, median (range) in years	57 (19–83)	57 (19–83)	59 (23–77)	0.609
Sex, number (%)				0.669
Male	105 (44.1)	86 (44.8)	19 (41.3)	
Female	133 (55.9)	106 (55.2)	27 (58.7)	
Tumor entity, number (%)				0.451
Stomach cancer	70 (29.4)	57 (29.7)	13 (28.3)	
LAMN	50 (21.0)	42 (21.9)	8 (17.4)	
Colorectal cancer	40 (16.8)	29 (15.1)	11 (23.9)	
Mesothelioma	29 (12.2)	21 (10.9)	8 (17.4)	
Ovarian cancer	18 (7.6)	17 (8.9)	1 (2.2)	
Appendix carcinoma	12 (5.0)	10 (5.2)	2 (4.3)	
Others	19 (8.0)	16 (8.3)	3 (6.5)	
Comorbidites, number (%)				
Arterial hypertension	77 (32.5)	60 (31.4)	17 (37.0)	0.471
Diabetes mellitus	16 (6.8)	12 (6.3)	4 (8.7)	0.558
Coronary artery disease	13 (5.5)	10 (5.2)	3 (6.5)	0.731
Renal disease	14 (5.9)	14 (7.3)	0 (0.0)	0.058
Body mass index, median (range) in kg/m^2^	24.0 (14.8–44.8)	24.1 (14.8–44.8)	23.6 (16.8–44.1)	0.893
Preoperative chemotherapy, number (%)	150 (63.3)	119 (62.3)	31 (67.4)	0.520
ASA classification, number (%)*^a^				0.221
1	11 (4.7)	9 (4.8)	2 (4.3)	
2	97 (41.3)	84 (44.4)	13 (28.3)	
3	124 (52.8)	94 (49.7)	30 (65.2)	
4	3 (1.3)	2 (1.1)	1 (2.2)	
PCI, median (range)	12.0 (0–39)	11.0 (0–39)	12.5 (0–39)	0.682
CCR, number (%)*^b^				0.174
0–1	169 (77.5)	139 (79.4)	30 (69.8)	
2–3	49 (22.5)	36 (20.6)	13 (30.2)	
Duration surgery, median (range) in minutes	394 (98–765)	389 (98–765)	426 (162–763)	0.190
Duration HIPEC, median (range) in minutes	90 (30–90)	60 (30–90)	90 (66–90)	**< 0.001**
Doxorubicin, number (%)*^c^	52 (21.9)	41 (21.4)	11 (23.9)	0.706
Mitomycin C, number (%)*^c^	185 (78.1)	150 (78.1)	35 (76.1)	0.765

The two-sided significance level was set to *p* < 0.05

*STS* sodium thiosulfate, *LAMN* low-grade appendiceal mucinous neoplasm, *ASA* physical status according to the classification system of American Society of Anesthesiologists,^32^*PCI* peritoneal cancer index, *CCR* completeness of cytoreductive surgery,^18^*HIPEC* hyperthermic intraperitoneal chemotherapy

*^a^Three patients excluded

*^b^Twenty patients excluded

*^c^One patient excluded because of missing information

### Intraoperative Characteristics

There was a significant difference in the duration of HIPEC between the STS and the no STS group due to the aforementioned change of protocol in January 2019, with prolonged HIPEC duration in the STS group. Apart from this, there were no significant differences between both groups regarding completeness of cytoreduction (CCR), duration of surgery, and the combined chemotherapeutic agent during HIPEC. Please refer to Table 1 for detailed information on intraoperative characteristics.

STS caused mild hypernatremia. There was a median increase in serum sodium of 5 mmol/l (range 1–12 mmol/l) following the STS bolus. In patients who received continuous STS in the intended dose, further median increase in serum sodium level was 3.0 mmol/l (range 1–6 mmol/l). Ten patients (21.7%) either did not receive the continuous administration of STS or received a reduced dose of continuous STS owing to hypernatremia. No patient demonstrated neurological symptoms or other secondary complications of hypernatremia, and none of the patients needed a specific treatment for hypernatremia.

### Postoperative Outcome Parameters

Hospital length of stay (LOS) was comparable between the STS and the no STS group (10 vs. 11 days; *p* = 0.741), whereas the median ICU LOS was significantly prolonged in the STS as compared with the no STS group (2 vs. 1 day(s); *p* = 0.031).

There was no statistically significant difference in the number and severity of postoperative complications classified according to Clavien–Dindo.^19^

For detailed information on postoperative outcome parameters please refer to Table 2.

**Table 2:** Postoperative outcome parameters after CRS and HIPEC

	All patients (*N* = 238)	No STS (*N* = 192)	STS (*N* = 46)	*p* value
AKIN stage, number (%)				**0.009**
0	176 (73.9)	133 (69.3)	43 (93.5)	
1	32 (13.4)	30 (15.6)	2 (4.3)	
2	17 (7.1)	16 (8.3)	1 (2.2)	
3	13 (5.5)	13 (6.8)	0 (0.0)	
AKI (AKIN ≥ 1),number (%)	62 (26.1)	59 (30.7)	3 (6.5)	**0.001**
Hemodialysis, number (%)	6 (2.5)	6 (3.1)	0 (0.0)	0.225
LOS hospital, median (range) in days	10 (3–163)	11 (3–163)	10 (5–62)	0.741
LOS ICU, median (range) in days	1 (0–153)	1 (0–153)	2 (1–35)	**0.031**
Clavien–Dindo classification, number (%)*^a^				0.359
0	88 (38.3)	73 (38.0)	15 (32.6)	
1	26 (11.3)	25 (13.0)	1 (2.2)	
2	51 (22.2)	38 (19.8)	13 (28.3)	
3a	24 (10.4)	20 (10.4)	7 (15.2)	
3b	25 (10.9)	21 (10.9)	6 (13.0)	
4a	9 (3.9)	9 (4.7)	2 (4.3)	
4b	7 (3.0)	6 (3.1)	2 (4.3)	
5	0	0	0	

The two-sided significance level was set to *p* < 0.05

*STS* sodium thiosulfate, *GFR* glomerular filtration rate, *AKIN stage* severity of acute kidney injury classified according to the Acute Kidney Injury Network,^16^*LOS* length of stay, *ICU* intensive care unit, *Clavien–Dindo* postoperative complications classified according to Clavien–Dindo^19^

*^a^Eight patients excluded because of missing information

### Preoperative and Postoperative Renal Function

There were no significant differences in preoperative serum creatinine, serum urea, and glomerular filtration rate between patients in the STS and no STS group.

Postoperatively, the incidence of AKI was significantly lower in the STS group when compared with the no STS group (6.5% vs. 30.7%; *p* = 0.001, Table 2). Within the patients with AKI, severity according to AKIN stages was significantly lower in the STS group. No patient in the STS group required renal replacement therapy following surgery compared with six patients (3.1%) in the no-STS group. This numeric difference did not reach statistical significance (*p* = 0.225). Please see Table 2 for detailed information on the postoperative renal function parameters.

**Fig. 3.**
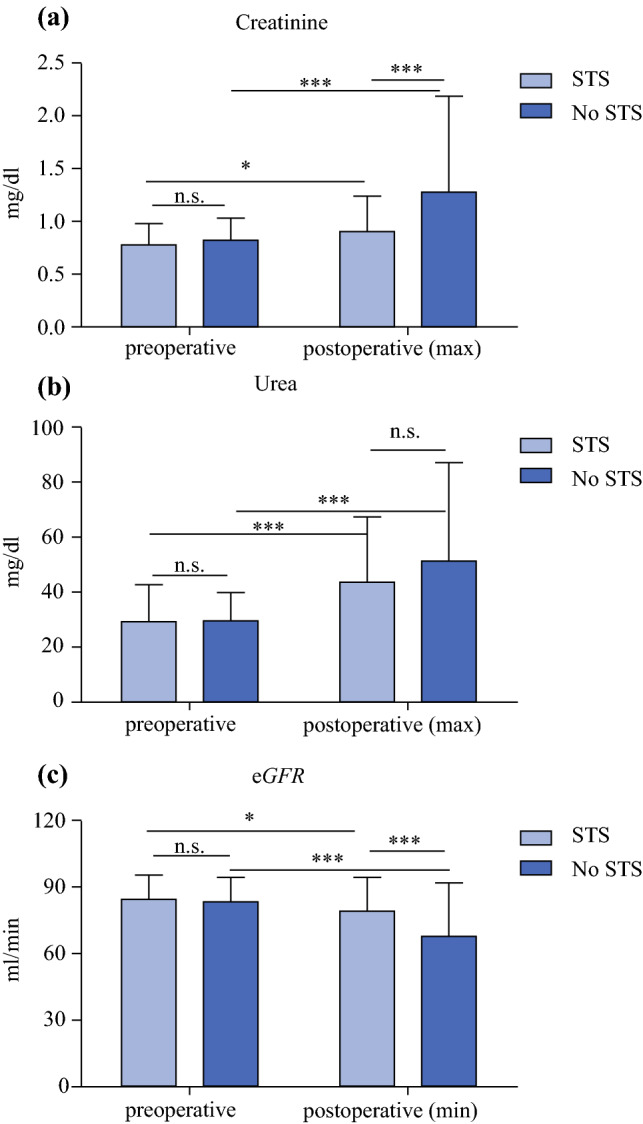
Laboratory kidney function parameters before and after CRS and HIPEC. Pre- and postoperative serum levels of creatinine (**a**), urea (**b**), and estimated glomerular filtration rate (eGFR, **c**). *STS* sodium thiosulfate

Maximum postoperative serum concentration of creatinine was lower in the STS versus the no STS group (0.87 mg/dl vs. 0.97 mg/dl, *p* = 0.004) with a corresponding higher minimum postoperative eGFR (88 ml/min vs. 77 ml/min, *p* = 0.007). In relation to preoperative baseline values, postoperative creatinine was significantly increased, and eGFR decreased in both groups, but the developments were more pronounced in the no STS group. Serum urea was significantly increased postoperatively in our cohort without a significant difference between the STS and the no-STS group at any time point (Fig. 3).

Of note, when excluding patients with preexisting renal disease from the analysis (*N* = 14), the differences in postoperative renal function parameters between both groups remain significant (*p* = 0.001). Similarly, when excluding all patients who had received 60 min of HIPEC instead of 90 min, the no STS group (*N* = 78) still had a significantly higher rate of AKI than the STS group (35.9% vs. 6.5%, *p* < 0.001).

### Risk Factors for Postoperative Acute Kidney Injury

To identify factors influencing postoperative renal function, patients were grouped according to the diagnosis of postoperative AKI defined as AKIN stage ≥ 1. Univariable analysis of baseline patient and tumor characteristics as well as intraoperative characteristics demonstrated that patients with AKI were significantly more likely to be male, at an advanced age, overweight, and affected by previous illnesses, such as arterial hypertension, diabetes mellitus, and coronary artery disease. Furthermore, preoperative laboratory parameters indicating impaired renal function (elevated serum creatinine and serum urea, reduced eGFR) were associated with a higher risk to develop postoperative AKI. Please refer to Table 3 for univariable analysis of potential risk factors for postoperative AKI.

**Table 3:** Univariable analysis of potential risk factors for postoperative AKI after CRS and HIPEC

	AKIN 0 (*N* = 176)	AKIN ≥ 1 (*N* = 62)	*p* value
Advanced age (≥ 60 years)	61 (34.7)	35 (56.5)	**0.003**
Sex, number (%)			**0.048**
Male	71 (40.3)	34 (54.8)	
Female	105 (59.7)	28 (45.2)	
Tumor entity, number (%)			0.835
LAMN	34 (19.3)	16 (25.8)	
Appendix carcinoma	8 (4.5)	4 (6.5)	
Colorectal cancer	29 (16.5)	11 (17.7)	
Stomach cancer	54 (30.7)	16 (25.8)	
Ovarian cancer	13 (7.4)	5 (8.1)	
Mesothelioma	22 (12.5)	7 (11.3)	
Others	16 (9.1)	3 (4.8)	
Comorbidities, number (%)			
Arterial hypertension	40 (22.9)	37 (59.7)	**< 0.001**
Diabetes mellitus	8 (4.6)	8 (12.9)	**0.025**
Coronary artery disease	5 (2.9)	8 (12.9)	**0.003**
Renal disease	8 (4.6)	6 (9.7)	0.143
Overweight (Body mass index ≥ 25 kg/m^2^)	70 (39.8)	34 (54.8)	**0.040**
Preoperative chemotherapy, number (%)	117 (66.5)	33 (54.1)	0.084
ASA classification, number (%)*^a^			0.232
1	10 (5.8)	1 (1.6)	
2	72 (41.6)	25 (40.3)	
3	90 (52.0)	34 (54.8)	
4	1 (0.6)	2 (3.2)	
Advanced peritoneal cancer index (PCI ≥ 15	66 (38.2)	30 (50)	0.108
STS, number (%)	43 (24.4)	3 (4.8)	**0.001**
CCR, number (%)*^b^			0.945
0–1	125 (77.6)	44 (77.2)	
> 1	36 (22.4)	13 (22.8)	
Extended duration of surgery (≥ 400 min)	79 (44.9)	36 (58.1)	0.074
Extended duration of HIPEC (≥ 60 min)	93 (52.8)	31 (50)	0.700
Doxorubicin, number (%)*^c^	39 (22.2)	13 (21.0)	0.845
Mitomycin C, number (%)*^c^	136 (77.3)	49 (79.0)	0.775
Elevated preoperative creatinine (≥ 1.0 mg/dl)	18 (10.3)	17 (27.4)	**0.001**
Elevated preoperative urea (≥ 45 mg/dl)	4 (2.3)	10 (17.2)	**< 0.001**
Reduced preoperative eGFR (< 90 ml/min)	60 (34.3)	36 (58.1)	**0.001**

The two-sided significance level was set to *p* < 0.05

*STS* sodium thiosulfate, *LAMN* low-grade appendiceal mucinous neoplasm, *ASA* physical status according to the classification system of American Society of Anesthesiologists,^32^*PCI* peritoneal cancer index, *CCR* completeness of cytoreduction,^18^*HIPEC* hyperthermic intraperitoneal chemotherapy, *LOS* length of stay, *ICU* intensive care unit, *Clavien–Dindo* postoperative complications classified according to Clavien–Dindo,^19^*GFR* glomerular filtration rate

*^a^Three patients excluded

*^b^Twenty patients excluded

*^c^One patient excluded

*^d^Eight patients excluded because of missing information

**Fig. 4.**
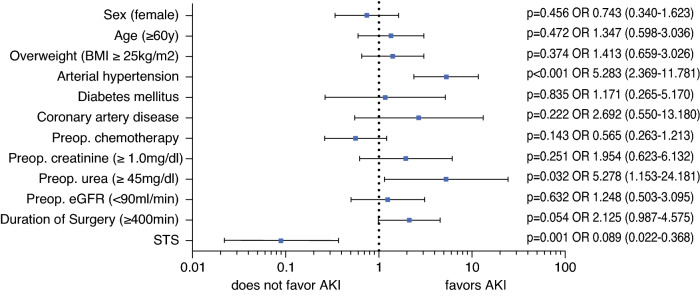
Multivariate analysis of potential risk factors for postoperative acute kidney injury (AKI) following CRS and HIPEC. *OR* odds ratio (95% confidence interval), *BMI* body mass index, *Preop.* preoperative, *eGFR* estimated glomerular filtration rate

On multivariate regression analysis including all preoperative and intraoperative factors that were associated with postoperative AKI in univariable analysis (*p* < 0.1), arterial hypertension (OR 5.283, *p* < 0.001), preoperative urea serum level (OR 5.278, *p* = 0.032), and the use of STS (OR 0.089, *p* = 0.001) remained independent predictive factors (Fig. 4). An extended duration of surgery (longer than 400 min) showed a trend towards a higher risk for AKI, but did not reach statistical significance (OR 2.125, *p* = 0.054).

As AKIN stage 1 could be argued to be of low clinical significance, we also performed a multivariate analysis for AKIN stage ≥ 2 including the same potential risk factors (Supplementary Fig. 1). The results are similar to those of the analysis using AKIN stage ≥ 1 as a cut-off. The only difference is that preoperative urea serum level in AKIN stages ≥ 2 no longer represents a significant risk factor for AKI.

## Discussion

The objective of this study was to elucidate to which extent perioperative STS administration protects patients with peritoneal surface malignancy undergoing CRS and CDDP-based HIPEC from postoperative AKI. Additionally, subgroup analyses were performed to identify contributing factors for postoperative renal function.

On analysis, the incidence of AKI in the overall cohort was 26.1%, which is comparable with the rates of AKI following CRS and CDDP-based HIPEC reported in current literature.^4^–^8^ Patients receiving STS demonstrated a lower incidence and also a lower severity of AKI than those without STS. None of the patients who received STS required renal replacement therapy compared with six patients in the no STS group (n.s.). Furthermore, on multivariate analysis including all factors that were associated with postoperative AKI on univariable analysis, the use of STS remained an independent predictive factor for unimpaired postoperative renal function. Importantly for the clinical relevance of our findings, the results of this multivariate analysis remain significant when using AKIN stage 2 as cut-off for the definition of AKI.

Baseline and intraoperative characteristics including preoperative laboratory renal function parameters were similar between the STS and the no STS group. Due to a change of our HIPEC protocol in January 2019, duration of HIPEC changed from 60 to 90 min, resulting in a significant difference in HIPEC duration in both groups. To control for this potential confounding factor, we performed a subgroup analysis comparing only patients who had received 90 min of HIPEC and obtained the same results with regard to incidence of postoperative AKI.

Our findings are endorsed by the results of a recent study by Laplace et al., who report an AKI incidence of 0% of STS and 31.4% of no STS patients (*p* < 0.05). Similar to our results, no patient in the STS-receiving population required renal replacement therapy.^15^ Tilleman et al. administered STS in patients undergoing extrapleural pneumonectomy followed by hyperthermic intrathoracic chemotherapy with CDDP 225 mg/m^2^ for malignant pleural mesothelioma.^22^ Despite the relatively high CDDP dose administered, AKI of any degree appeared in only 10.8% of patients.^22^ These findings support the proposal that STS might serve as an effective kidney-protective strategy in patients undergoing CRS and CDDP-based HIPEC.

Apart from the identification of kidney-protective measures, it seems reasonable to search for additional factors influencing AKI in patients treated with CRS and HIPEC. While male gender, advanced age (> 60 years), overweight, arterial hypertension, diabetes mellitus, coronary artery disease, and preoperative laboratory parameters indicating impaired renal function were significantly associated with postoperative AKI on univariable analysis, only arterial hypertension remained an independent risk factor on multivariable analysis. These results are partly consistent with those of an observational study identifying preexisting comorbidities classified according to the Charlson Comorbidity Index as factors significantly associated with AKI following CRS and HIPEC.^5^ In another retrospective study including 475 patients, the authors identified not only advanced age and obesity, but also male gender as risk factors for the development of AKI following CRS and HIPEC.^4^ These findings indicate that there might be a relevant percentage of preexisting, nondiagnosed renal damage in patients with the aforementioned baseline characteristics and comorbidities. It can be concluded that these patients represent a high-risk population with the need for effective kidney-protective strategies and a close monitoring of renal function parameters following CRS and HIPEC.

Apart from patient-specific factors, treatment strategies beyond surgery, especially perioperative fluid management, might have an impact on the incidence of postoperative AKI. In recent years, standards for perioperative care, such as enhanced recovery after surgery (ERAS) guidelines, have been promoted to reduce the length of hospital stay, costs, and complication rates.^23^^,^^24^ First published studies in CRS and HIPEC patients show some promising results; however, there was no impact on AKI rates.^25^–^27^ In our own cohort, fluid management was guided by our SOP for all patients (STS or no STS) and was restrictive after the day of surgery.

On analysis, neither hospital length of stay nor postoperative complication rates differed significantly between patients receiving STS and those not receiving STS, which might be due to the small number of patients developing severe postoperative complications following CRS and HIPEC. The same observation was made by Laplace et al.^15^ The authors did not identify significant differences in the rates of surgical and medical complications other than nephrotoxicity between patients with or without STS administration. However, patients in the STS group stayed a median of two nights in the intensive care compared with one night in the no STS group. Due to the experimental administration of STS based on insufficient clinical evidence in this patient cohort, we exercised caution when hypernatremia as a well-known side effect of STS occurred, resulting in a prolonged ICU stay for the purpose of a close monitoring of electrolyte imbalance until a normalization of serum sodium concentration. This is underlined by the fact that we neither observed complications from hypernatremia nor any specific treatment was required for hypernatremia in our patients.

The occurrence of AKI in our cohort was significantly associated with a prolonged hospital stay. This is in accordance with the results of a previous study reporting on secondary complications caused by renal injury following CRS and HIPEC.^5^ These data underline the importance of avoiding postoperative AKI and consequently reducing the risk for the development of chronic kidney disease with the corresponding implications for subsequent antitumor treatment.

In our study, we observed no side effects from the administration of STS apart from mild hypernatremia. This is in line with previous studies reporting that side effects of STS in humans are rare and mainly grade 1 or 2 according to Common Terminology Criteria for Adverse Events.^9^^,^^10^^,^^12^^,^^15^

In preclinical animal models, STS was not shown to affect antitumor efficacy of CDDP.^28^–^31^ Further research is needed to reliably exclude an impact of STS on antitumor activity.

Limitations of the present study are the retrospective nature of the analysis, and the limited number of patients in the analyzed subgroups. Additionally, patients in the STS and no STS group were treated in two successive time intervals introducing potential further bias. However, apart from a differing duration of HIPEC due to protocol changes, there were no significant differences in baseline and perioperative characteristics between both groups. Strengths of our study include the highly standardized treatment of patients in our center with a small team of specialized surgeons and defined internal standard operating procedures, as well as the robust results of a multivariate analysis of numerous potential predictive factors for postoperative AKI.

## Conclusions

In accordance with recent studies, our data strongly suggest that STS effectively protects from CDDP-induced acute renal injury in patients undergoing CRS and HIPEC. Taking into account the controversial data on the benefit of CRS and HIPEC in certain tumor entities and treatment settings, there is an urgent need to prevent complications, such as AKI, which is known to be associated with a prolonged hospital stay and further major morbidities.^5^

Future prospective randomized studies will be needed to validate the benefit of STS among kidney-protective strategies. These studies should also focus on the identification of additional risk factors for AKI following CRS and HIPEC to better define high-risk patients before surgery.

## Supplementary Information

Below is the link to the electronic supplementary material.

Supplementary file1 (PDF 1680 kb)
