# Supplementary material for: Sodium Thiosulfate Reduces Acute Kidney Injury in Patients Undergoing Cytoreductive Surgery Plus Hyperthermic Intraperitoneal Chemotherapy with Cisplatin: A Single-Center Observational Study
Source: Ann Surg Oncol. 2021 Aug 4;29(1):152–62. doi: 10.1245/s10434-021-10508-x (PMC8677645; doi:10.1245/s10434-021-10508-x)
Supplement: Supplementary file 1 — Supplementary file1 (PDF 1680 kb) [file 10434_2021_10508_MOESM1_ESM.pdf]

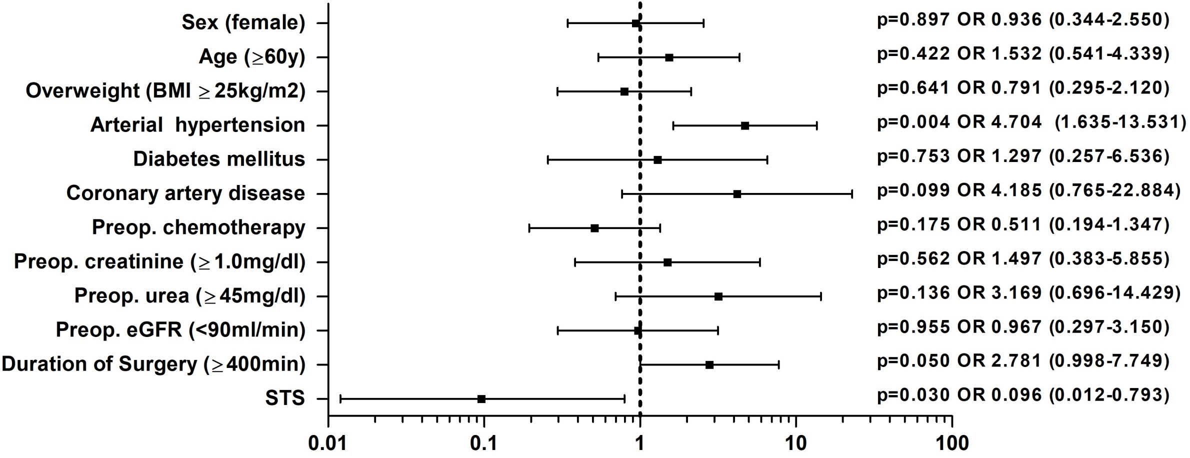

SUPPLEMENTARY FIGURE 1: Multivariate analysis of potential risk factors for postoperative acute kidney injury (AKI) AKIN stage ≥2 following CRS and HIPEC. OR - Odds Ratio (95% confidence interval); BMI- body mass index; Preop.- preoperative; eGFR- estimated glomerular filtration rate.
